# Oculomotor axons use external and autocrine Slit signals for fasciculation, navigation, branching, and connectivity in mouse embryos

**DOI:** 10.21203/rs.3.rs-5718241/v1

**Published:** 2025-05-13

**Authors:** Claudia M. Garcia-Pena, G. Eric Robinson, Minkyung Kim, J. Sterling Louw, Chase Johnson, Brielle Bjorke, Grant S. Mastick

**Affiliations:** University of Nevada Reno

**Keywords:** Slit, Robo, motor neuron, oculomotor development, axon guidance, autocrine, eye muscle innervation

## Abstract

**Background:**

Eye alignment and movements are controlled by three cranial nerves: the oculomotor (nIII), trochlear (nIV), and abducens nerves (nVI). Developmental errors of these nerves can lead to eye movement disorders. However, the molecular and cellular mechanisms that guide motor axons to the eye remain poorly understood.

**Methods:**

Oculomotor nerve guidance was examined in mutant mouse embryos for the secreted Slit proteins and Robo receptors. Methods included mapping Slit and Robo expression using Slit transgenic markers and Robo antibodies, imaging of oculomotor nerves and muscles using antibody labeling, and culture of oculomotor axons with Slit proteins.

**Results:**

Robo1 and 2 were expressed in motor axons, while Slits were expressed surrounding the motor pathway, in mesenchymal tissues around the eye muscles, and by motor axons. Robo1/2 or Slit1/2 mutant embryos had similar guidance defects at several steps. The initial nerves projecting out to the eye were defasciculated. Upon reaching the eye, oculomotor axons normally stop to form a plexus in contact with muscle precursor cells, but in Robo and Slit mutants, the motor axons spread abnormally to overshoot. Later, abnormal nerve branches resulted in reduced muscle innervation. Importantly, motor neuron-specific deletion of Slit2 caused a subset of these errors.

**Conclusions:**

Several steps along the navigational pathway of oculomotor nerves are controlled by Slit/Robo signals, including fasciculation, correct navigation to an intermediate target, and innervation of extraocular muscle. Furthermore, important aspects of motor axon guidance are controlled by autocrine Slit2 signals produced by motor axons themselves.

## Background

During brain development, motor axons extend to their targets in the periphery by navigating through several steps. First, pioneer axons exit the brain, to navigate along stereotyped pathways. These initial motor axons bundle together tightly into fasciculated motor nerves. Secondly, motor axons pause in a plexus or decision point, prior to muscle differentiation (Tosney and Landmesser, 1985). Finally, motor axon growth resumes by subpopulations of axons splitting off from the main nerves to form branches that approach and penetrate specific muscles. However, whether distinct signals guide these steps in motor axon navigation remains poorly understood.

The motor nerve projections to the eye develop early, with many aspects of functional organization and molecular development evolutionarily conserved (Fritzsch, 2024; Whitman and Engle, 2017). The main nerve is the oculomotor nerve (nIII), innervating four of the six extraocular muscles (EOM), while the trochlear (nIV) and abducens (nVI) nerves each innervate one muscle. The steps of oculomotor axon navigation include projecting in a tight bundle from midbrain to the eye (Mastick and Easter, 1996). The axons pause near the eye for several days in a plexus in contact with precursor cells (Bjorke et al., 2021). Then the nIII extends branches to differentiating muscles (Bjorke et al., 2021; Michalak et al., 2017). These simple nerve patterns have the potential to facilitate identification of molecular guidance cues.

There are likely multiple tissues and molecular signals that guide specific steps in oculomotor nerve guidance. A forward genetic screen in mouse embryos for cranial nerve errors found several mutants with defects most commonly in the oculomotor nerve (Mar et al., 2005). The nIII plexus near the eye interacts with Pitx2-expressing precursors, with axons failing to pause after precursor ablation (Bjorke et al., 2021). Similarly, loss of eye muscles causes loss of oculomotor branches (Michalak et al., 2017). Other guidance cues are implicated by expression patterns and nerve errors in mouse mutants. Several Semaphorin family members are expressed around the developing chick eye muscles, while their Neuropilin (Npn) receptors 1 and 2 are expressed in the oculomotor nucleus (Chilton and Guthrie, 2003). On the functional level, mouse mutants for either Sema3F (Sahay et al., 2003) or Npn2 (Chen et al., 2000) disrupt the outgrowth of nIII by causing defasciculation, and loss or reduction of nIV projections toward the eye. Human mutations in the intracellular adaptor a2-chimerin, a mediator of Sema/Npn signals, result in Duane’s syndrome, involving nVI errors (Carretero-Rodriguez et al., 2021; Clark et al., 2013; Miyake et al., 2008; Whitman and Engle, 2017). Muscle-derived growth factors SDF/HGF were also implicated in nerve attraction to chick eye muscles (Lerner et al., 2010).

The Slit/Robo guidance cue system has also been implicated in oculomotor nerve guidance. Slit2 and the Slit receptors Robo1 and 2 are expressed by motor neurons (Brose et al., 1999), including the nIII, nIV and nVI nuclei (Hammond et al., 2005). Slit2 is implicated as a motor axon-derived autocrine/paracrine signal that promotes fasciculation of spinal motor axons in culture, and Slit2 mutants have phrenic nerve defasciculation (Jaworski and Tessier-Lavigne, 2012). The oculomotor nucleus similarly expresses Slit2 and 3, and both Robo1 and 2 (Bjorke et al., 2016). Slits and Robos function to prevent premature contralateral migration of oculomotor neuron cell bodies across the midbrain floor plate (Bjorke et al., 2016).

In this study, we test the function of Slit/Robo signals to guide oculomotor axons to the eye. We studied oculomotor nerve development in Slit or Robo mutant mouse embryos, including motor-neuron specific deletion of Slit2. We found that Slit/Robo signals are required to guide motor nerves at several steps, including by promoting nerve fasciculation, forming a plexus at the correct position, and guiding branches accurately to muscles. Furthermore, several of these Slit2-dependent steps require motor neuron production of Slit2, evidence for function as an autocrine guidance signal in vivo. Overall, our studies reveal that Slit/Robo signals are required in distinct ways at several decision points in oculomotor nerve development.

## Methods

### Animals

Mouse experiments were done using the UNR IACUC protocol 00435 (21-01-1123), following NIH guidelines. The Robo and Slit mutant founder mice were gifts of Marc Tessier-Lavigne (Stanford; Genentech) (Long et al., 2004). Islet-1MN-GFP-F mice were gifts of Samuel Pfaff (Salk Institute) (Lewcock et al., 2007). Embryos from E10.5 to E14.5 mutant embryos were obtained by mating heterozygotes, using the breeding stocks of single Robo1^+/−^, single Robo2^+/−^, combined Robo1^+/−^; Robo2^+/−^, combined Slit1^−/−^; Slit2^+/−^, or combined Slit1^+/−^; Slit2^+/−^. Littermates were compared for analysis to minimize differences in developmental stages. For motor neuron-specific deletions, Slit2^flox^ (Rama et al., 2015) and Robo2^flox^ (Lu et al., 2007) (gift from Chedotal A, Vision institute and Ma L from Jefferson University) were crossed with Islet-CRE (gift from Tom Gould, University of Nevada, Reno) to obtain a specific deletion of Slit2 or Robo2 in motor neurons. We previously validated that motor neuron-specific deletion by this strategy depleted Slit2 mRNA signal by in situ hybridization in motor neurons, but not in the adjacent floor plate (Kim et al., 2019).

Embryos were obtained by uterine dissection followed by PCR genotyping from a limb or tail sample.

### Preparation of samples: bisected embryos, sectioning with vibratome and cryostat

Intact embryo heads from E10.5, or bisected heads from E11.5 were immuno-stained whole, as described in the next section. E12.5 and E14.5 heads were prepared for sectioning by vibratome by first washing embryo heads three times for 1hr in 0.1 M phosphate buffer (pH 7.4). Then, bisected heads were positioned in 1% melted agarose in Tissue-tek cryomolds (4557, 25mm ×20mm × 5mm) at room temperature until solidification. For sagittal or coronal sections, the agarose blocks were fixed with super glue on the Vibratome platform, then 400 microns sections were cut. Sections were washed three times with 4 ml of 1X PBS in 10ml glass vials.

For cryostat sections, additional samples were immersed in 5% sucrose/0.1M PO4 for 1 hr at room temperature (RT), followed by 15%sucrose/0.1M PO4 overnight at 4C degrees on a rotator. The next day, samples were incubated in 7.5% gelatin, 15% sucrose in 0.1M PO4 in a 37°C incubator for 24 hours. Samples were positioned in a mold created with foil, then immersed in a dry ice/methyl butane bath to freeze. Using a Leica Cryostat, 30 microns sections were cut and placed on Fisher Superfrost slides, and stored at −20C.

### Immunostaining

The immunostaining procedure for vibratome sections or bisected embryos was based on the IDISCO protocol (Renier et al., 2014), with specific variations to improve the penetration of antibodies. Samples were washed 3 times for one hour in PBS (pH 7.5) including 0.02% sodium azide (PBS1X) at room temperature. The samples were dehydrated with a methanol/PBS1X series, consisting of one hour each in 50% and 80% methanol, and two times in 100% methanol for 1 hour, then overnight in 100% methanol at 4°C. Next day, samples were washed in bleaching solution consisting of methanol, 20% DMSO and 5% peroxide overnight at 4°C. Samples were washed at room temperature with 100% methanol, 3 times for 1 hour each. Samples were incubated 2 hours with Methanol 100% and 20% DMSO. Rehydration was performed by decreasing concentrations of methanol (80%, 50%, and 0%) 1 hrs in PBS1x. After the last wash in PBS1X, permeabilization was performed with 0.2% Triton 100X in PBS1X, twice for one hour. The first blocking step was overnight at 37°C in PBS1X including 0.2% Triton, 20% DMSO and 0.3M glycine (BDH). A second blocking step was performed with PBS1X, 0.2% Triton, 10% DMSO, and 5% goat serum for two days at 37°C. Three washes of 1 hour at room temperature with PBS1X (pH 7.4), 0.2% Tween-20, and 10 mg/ml of heparin (PTwH). Incubation with diluted primary antibody was 5–7 days at 37°C. After 5–7 days, the antibody was washed 5 times for 1 hour with PTwH at room temperature on a rotator, continuing with an overnight wash. After a final wash with PTwH for 1 hour, samples were incubated with secondary antibodies for 5–7 days at 37°C. Samples were then cleared by incubation in 80% glycerol, and mounted in imaging chambers.

The antibodies used were: anti β-III-tubulin made in rabbit (used at 1:2000; 802001, Biolegend, San Diego, CA); anti β-III-Tubulin made in mouse (used 1:2000; 801202, Biolegend); anti-green fluorescent protein (GFP) made in rabbit (used at 1:1000; PA1980A, Invitrogen by Thermofisher, Waltham, MA); Actin α-smooth muscle made in mouse, as a Cy3 conjugate (used 1:2000; C6198, Sigma, St. Louis, MO); anti-Robo 1 made in rabbit (used 1: 500; gift from Elke Stein, Yale); anti-rat Robo 1, made in goat (used at 1:500; AF1749, R&D, Minneapolis, MN); anti-human Robo 2 made in goat (used at 1:400; AF3147, R&D).

The secondary antibodies used were: donkey anti-goat 488 (Invitrogen, Thermofisher); donkey anti-rabbit, Alexa 555 (Invitrogen); donkey anti-mouse Cy3 (Jackson); goat anti-rabbit Alexa 488 (Invitrogen). Each secondary antibody was used at 1:1000 dilution.

### Explant assay

Oculomotor nuclei of E11.5 Islet-1MN-GFP + embryos were dissected to exclude the floor plate under a fluorescence dissecting microscope (Leica M165 FC). Culture conditions for explants were as described previously (Charron et al., 2003). To assay directional effects, localized Slit2 source was provided as described in our previous paper (Kim et al., 2014). Explants were co-cultured with aggregates of COS-7 cells transfected with no plasmid (mock) or Slit2 full length expression plasmid (gift of Yi Rao, Beijing) for 48 hours. The length of axons was measured using the Image J plugin Neuron J (Kim et al., 2014). For the directional analysis, quadrants were marked on images using Adobe Illustrator (San Jose, CA), then imported and quantitated in Image J (NIH, Bethesda, MD). The outgrowth ratio was calculated by the average axon length in the quadrant toward the COS cells divided by the average axon length in the quadrant away from the cue source. To assay for fasciculation, oculomotor nuclei of E11 Islet-1MN-GFP-F were cultured for 48 hrs with mock-conditioned and Slit2-conditioned media. Axonal fasciculation was quantified using Image J by counting the number of axon bundles with a diameter over 8 microns.

### Imaging and Statistical analysis

Images were collected using a Leica TCS SP8 Confocal microscope using a 10x or 20x objective. Image stacks were 1024 × 1024 pixels, imaging to a depth of 400 microns with a Z resolution of 1.5 to 2 microns between slices. 3D images were reconstructed in FIJI Image J using the 3D viewer. In most images, a maximum Z projection is shown of the two or three channels. To highlight the nerve trajectories, the app ColorPop Microsoft Pro was used to manually color annotate specific nerves, as recently described (Bjorke et al., 2021). Briefly, images of maximum projections were color segmented by referring to Z stacks to trace the trajectory of a single nerve through the depth of the Z stack. This was effective in tracing nerve projections, even for nerves projecting near other tubulin-expressing nerves, such as the trigeminal ganglion. The color segmentation was done conservatively, with ambiguous nerve segments left uncolored, such as abnormal nerve contacts observed particularly in mutants with wandering or abnormal nerve branches. Therefore, some nerve errors are not shown in the images. To test the reproducibility of the color segmentation, this was done on several images by two independent people, and resulted in very similar color segments.

For statistical analyses, JASP software was used to perform one way ANOVA and t-tests to compare between groups, with numbers of samples indicated in the graphs or figure legends. Graphs were made with Graph Prism 8.

## Results

### Summary of time course of navigation of the oculomotor nerves.

**Figure 1.**
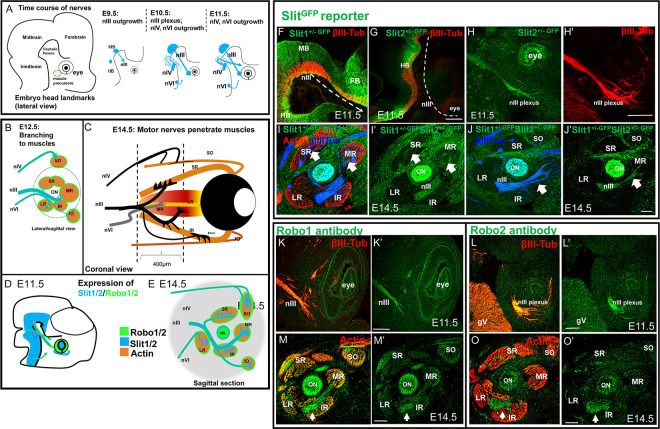
Slits and Robos are expressed in developing oculomotor nerves and their targets **A**) Schematic summary of the trajectories of the oculomotor nIII, trochlear nIV, and abducens nVI from E9.5 to E12.5 (lateral view). nIII grows out earliest as a tight fascicle from the midbrain, then pauses for two days in contact with muscle precursor cells, before branching out when muscles differentiate on E12.5. nIV exits next from anterior hindbrain, forming a less fasciculated nerve to the dorsal side of eye. nVI grows out later from the hindbrain to the lateral side of the eye. **B**) Schematic representation of nerve contacts with primordial muscles on E12.5, lateral/sagittal view. Muscle fibers appear in five clusters near the optic nerve (ON), while the superior oblique appears dorsally and inferior oblique ventrally. **C)** Coronal view (relative to the embryo head) on E14.5, showing the nerve branches to the six differentiated muscles. Dotted lines represent 400 microns sections in sagittal plane shown in E; yellow indicates optic nerve, with medial and lateral recti on either side. Motor nerves penetrate the muscles. nIII bifurcates to contact the superior rectus (SR) muscle, and the inferior rectus (IR) muscle. The inferior branch forms a tertiary branch that contracts the medial rectus (MR) muscle. A fourth branch from the nIII extends ventrally to the inferior oblique (IO). The nIV penetrates a specific region of the superior oblique (SO) muscle, and also forms a transitory branch that projects to the ventral region of the superior rectus. The nVI penetrates the lateral rectus (LR), nearly contacting the nIII. **D, E)** Schematic of Slit (blue) and Robo (green) expression, in lateral (sagittal) view on E11.5 and E14.5. This summarizes the expression patterns shown in panels 1F-T. Axons from all three nerves expressed Robo1 and 2, as well as Slit2 (shown as blue core with green shell). At E14.5, muscles expressed Robo1 at lower levels. Slit1 and Slit2 were expressed in the ventral neural tube flanking the nerve pathways, and in the area around the eye. **F-J)** Slit1 and 2 expression mapped by GFP reporters in the Slit knockout alleles, on E11.5 (F-H) and E14.5 (I-J). Slit2-GFP signal was visible above background levels, in the nIII (H, also nIV and nVI, not shown), and in the ventral forebrain, midbrain, and hindbrain. On E14.5, Slit1 and 2 GFP signal was visible in cells surrounding and between the muscles (I-J. white arrows). **K-O)** Expression of Robo1 and 2, mapped by antibody labeling on E11.5 (K-L) and 14.5 (M-O). Robo1 and 2 was expressed in the nIII nerve (white arrows, and also nIV and nVI, not shown). Abbreviations. nIII, oculomotor nerve; nIV, trochlear nerve; nVI, abducens nerve; TEL, telencephalon; MB, midbrain; HB, hindbrain; ON, optic nerve; gV, ganglion of the trigeminal nerve; SO, superior oblique muscle; SR, superior rectus muscle; LR, lateral rectus muscle; MD, medial rectus muscle; IR, inferior rectus muscle; IO, inferior oblique; bIII-tub, beta III tubulin. Scale bars represent 100μm.

**Figure 2.**
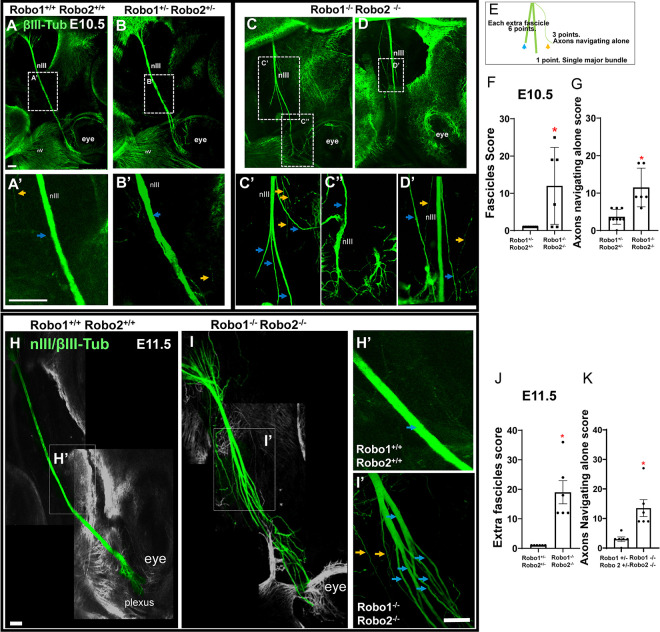
Robo 1 and 2 control cohesion of the oculomotor nerve. Oculomotor nerves in E10.5 (A-D) and E11.5 (H,I) embryos of wildtype and Robo1^+/−^2^+/−^ controls, and Robo1^−/−^2^−/−^ mutants were labeled with βIII-tubulin antibody. **A, B)** Robo1^+/+^ 2^+/+^ or Robo1^+/−^ 2^+/−^ controls. Close ups of controls (**A**’**B’)** showing the normal cohesion of nIII. Yellow arrows indicate a few single axons defasciculated from the main nerve, blue arrows, main bundle. **C,D)** Robo 1^−/−^;2^−/−^ mutant. **C’)** Several aberrant fascicles were observed (thickness <5μm) (blue arrows) Yellow arrows indicate axons navigating detached from the main nerve. **C”** shows a close up where the nerve stopped near to the eye, but formed an altered plexus. **D’)** Other alterations in Robo1^−/−^;2^−/−^ mutants. The plexus expanded, and some axons navigated far away from the main nerve (yellow arrows). **E)** Quantification of axon errors, where blue arrows indicate a score of 6 points for fascicles with thickness more than 5 μm. Yellow arrows indicate a score of 3 points for axons navigating alone, where the tip of the axon was located outside of the main bundle. The main nerve was given 1 point. **G)** Quantification of defasciculation. Comparison between controls (n=8) and Robo1^−/−^;2^−/−^ (n=6) at E10.5. In Robo1^−/−^;2^−/−^ mutants, the axons navigated alone more often than in controls. **H, H’)** E11.5, with this and later stages shown as projections of confocal z-stacks, with coloring of oculomotor nerve. Normal pattern of the cohesive nIII, with rarely more than one axon outside of the nerve. **I)** Robo1^−/−^;2^−/−^ defasciculated nerve. Yellow arrows in **H’** and **I’** indicate axons separated from the other fascicles. **J, K)** Quantification of E11.5 fasciculation shows similar errors in Robo1/2 double mutants. One way ANOVA was performed to compare between controls (n=6) and Robo double mutants (n=7), where p<0.05 in a Tukey post hoc test analysis was considered as significantly different. Scale bars, 100μm.

**Figure 3.**
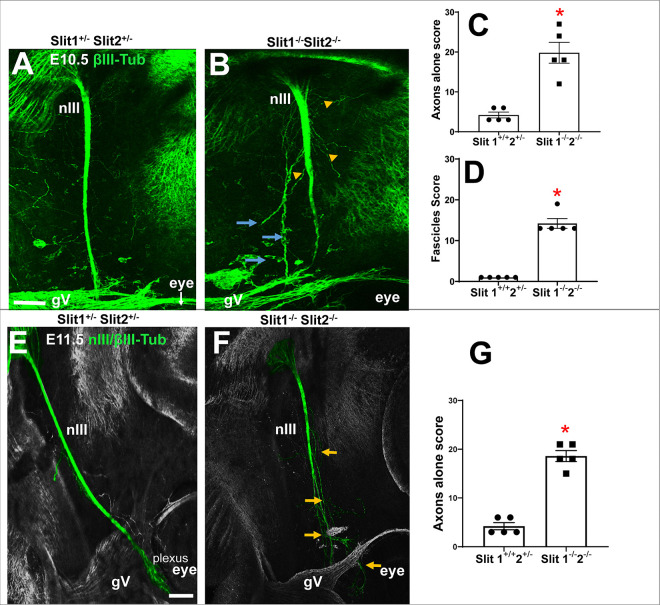
Slit 1 and 2 control fasciculation of the oculomotor nerve. Embryos at E10.5 (A-D) and E11.5 (E-G) were stained against βIII-tubulin to visualize nIII. **A)** Heterozygote E10.5 control had cohesive nIII projecting towards the eye. **B)** Slit1^−/−^; Slit2^−/−^ double mutant where the nIII formed extra fascicles (blue arrows), as well as axons navigating alone (yellow arrowhead). **E)** E11.5 nIII normal projection, where the nerve is reaching the area near the eye (green), typically with just one main fascicle and formation of a plexus. **F)** Slit1^−/−^; Slit2^−/−^ double mutant at E11.5. Several axons were defasciculated (arrows). **C, D and G)** Graphs of scores obtained from control and Slit1^−/−^; Slit2^−/−^ embryos, where double mutants formed significantly more fascicles and single axons detached from the nIII nerve. Five different mutants and controls were analyzed with one way ANOVA, with significant (p<0.05) increases in axons navigating alone in Slit mutants. Scale bars, 100μm.

**Figure 4.**
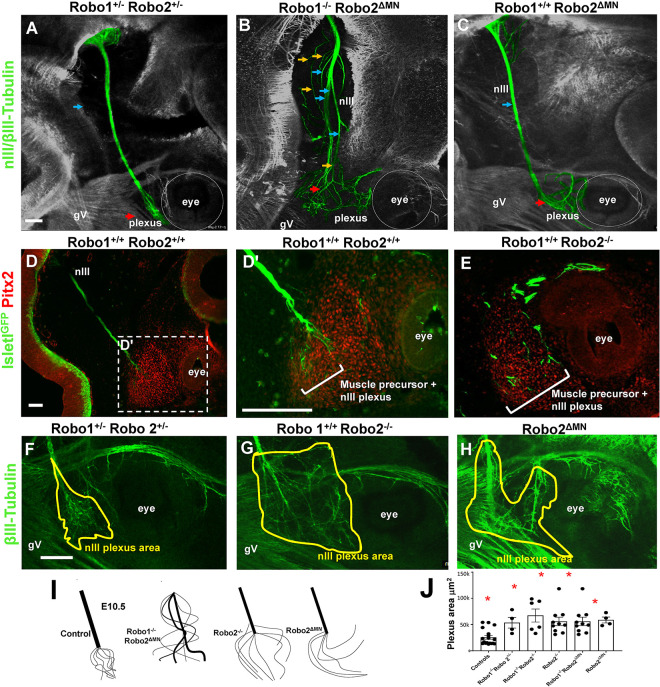
Robo2 autonomous function in motor axons controls fasciculation and plexus. To test motor neuron-specific knockouts of Robo2, whole mount E10.5 Robo1^+/−^; Robo2^+/−^ or Robo1^+/−^; Robo2^+/+^ (controls) and Robo1^−/−^;Robo2^ΔMN^ or Robo1^+/+^ Robo2^ΔMN^ were stained against β-III-tubulin. Shown are confocal z-stack projections. Although the β-III-tubulin marker was not specific for oculomotor nerves, nIII axons were clearly identifiable as a separate population in the confocal z-stacks, except in cases where the axons in mutants made abnormal contacts with other axons, such as trigeminal axons. Some genotypes were sectioned and stained for Pitx2 to visualize precursor cells. **A)** Robo1^+/−^; Robo2^+/−^ control embryo, with normal early projection of nIII where the nerve (blue arrow) projected toward the eye, ending in a plexus (red arrow). **B)** Motor neuron-specific deletion of Robo2 floxed allele in Islet1-Cre neurons (see Methods). Yellow arrows show axons navigating alone; blue arrows show the extra fascicles present; red arrow points to the plexus. **D, D’**) 20 micron thick cryostat sections showing plexus formation, with axons (green) in contact with Pitx2-expressing precursor cells (red). Loss of Robo function results in spreading of the plexus (E). **F-H)** Robo mutants show an expanded area of the plexus (yellow outline) in confocal images of whole mounts. **I)** Schematic representation of the plexus of each genotype. **J)** Quantification of axon spread within the plexus by area, with Robo mutants showing significant differences (one way ANOVA p<0.01) (n=4–10 for each genotype), with Tukey post hoc test performed to compare groups. Scale bars, 100μm.

**Figure 5.**
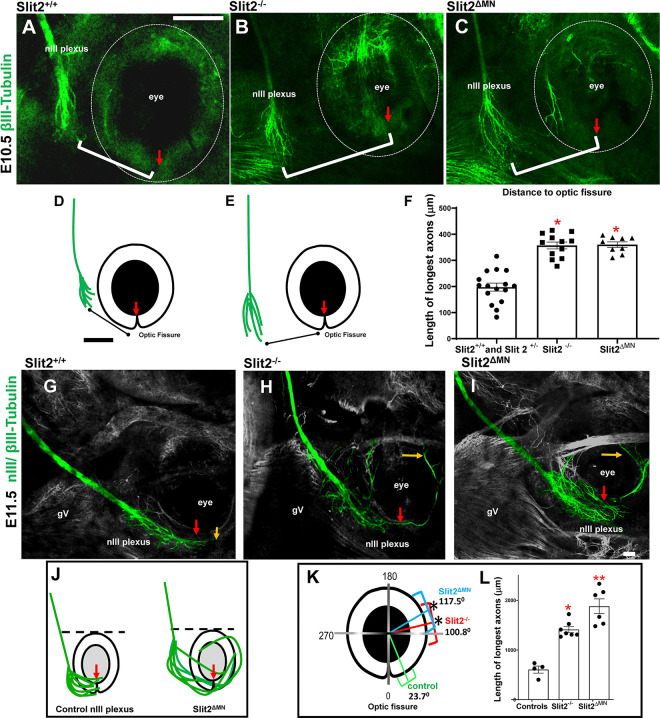
Motor axons require autonomous Slit2 both for correct trajectory and to stop near the eye. E10.5 and 11.5 embryos, labeled with b-III tubulin showing the position and size of the nIII plexus, relative to the eye, with images of single confocal planes. **A, B, C)** E10.5. Single confocal planes showing the nIII plexus. **B, C)** Both Slit2 knockout (Slit2^−/−^) and specific deletion of Slit2 in motor neurons (Slit2^ΔMN^) shifted the plexus away from the eye. **D, E)** Cartoons of control and Slit2^ΔMN^ respectively. **F)** Quantification of the distance of five longest axons to the optic fissure (red arrows, brackets in A,B,C). Slit2^ΔMN^ or Slit2^−/−^mutants caused a shift away from the eye (one way ANOVA, * = p>0.05). **G-I).** E11.5. In control embryos, the longest axons in the plexus reached close to the optic fissure. Oculomotor axons overshot the plexus in Slit2^−/−^ and Slit2^ΔMN^ mutants, with the longest axons (yellow arrows) overshooting the plexus to grow around the medial side of the eye **(H, I). J)** Cartoon summary of overshooting axons in Slit2^ΔMN^. **K, L)** Quantification of the position of the longest axon tips, represented as angles compared to the optic fissure. The brackets in K show the mean of the angles measured (control, green, Slit2−/− red, Slit2^ΔMN^, blue). **L)** Overshooting axons were longer in Slit2^−/−^ and in Slit2^ΔMN^ mutants. One way ANOVA test, p<0.05*, p<0.01** and Tukey post hoc tests. E10.5 controls, n=16; Slit2^−/−^, n=12; Slit2^ΔMN^, n=9. E11.5 controls, n=4; Slit2^−/−^, n=7; Slit2^ΔMN^, n=6. Left and right sides were quantified independently from each embryo. Scale bars, 100μm.

**Figure 6.**
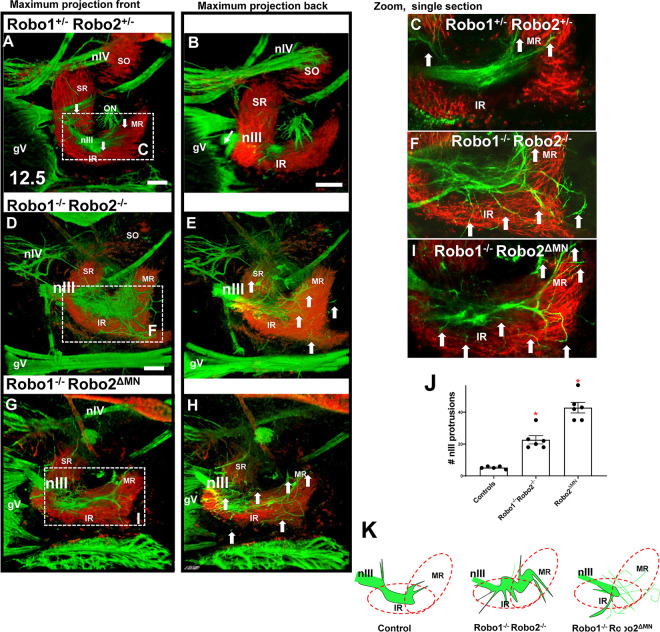
Robo1 and 2 control branches to extraocular muscles. E12.5 embryos labeled for βIII-tubulin to visualize axons (green) and actin as muscle marker (red). **A-C**) The control nIII branches formed compact dense projections that contacted the primordia of the medial, inferior and superior rectus muscles, with few stray axons visible (arrows in C). Panel B is a view from the back of the eye to show that the branches make contact in restricted regions of the mid-belly of the extraocular muscles. Relative to the anterior-posterior axis of the eye, the nerve branches are localized to the anterior (front) side of the muscles, in that they are visible in the front projection (A) but not the back projection (B). **D-F)** Robo1^−/−^;Robo2^−/−^ double mutant. The nIII was highly disorganized, with the main branches appearing less dense, and with axons shooting across and outside of the muscle. **E)** Mutants have numerous axons in the posterior part of the extraocular muscles (white arrows). **F** is a close up of **D**, showing stray axons that overshoot the muscles to project outside of the inferior rectus. **G-I)** Robo1^−/−^; Robo2^ΔMN^ embryos, with similar errors where many axons shot out of the area of the muscles (white arrows). **J)** To quantify the errors that disrupted branches to muscles, the number of protrusions from the nIII were counted in each genotype. E12.5 control, n=5; Robo1^−/−^; Robo2^−/−^, n=6; Robo1^−/−^; Robo2^ΔMN^, n=6. The nIII in Robo1^−/−^;Robo2^−/−^ and Robo1^−/−^;Robo2^ΔMN^ had a significant increase in aberrant protrusions (p<0.05). **K)** Summary of Robo1/2 mutant phenotypes. Scale bar 100μm.

**Figure 7.**
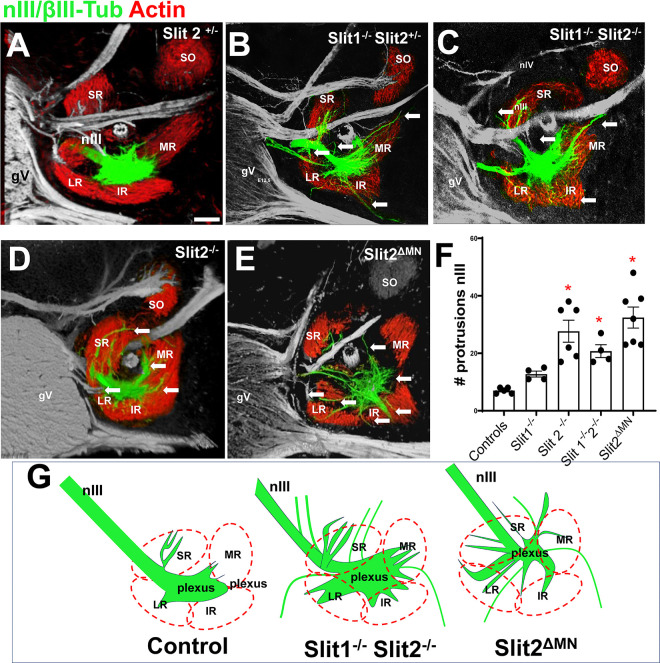
Slit signals control oculomotor nerve contact with the extraocular muscles. E12.5 embryos labeled with βIII-tubulin (green, selected just for nIII) and actin (muscle marker). **A)** Controls, Slit1^+/−^ or Slit2^+/−^, with compact nIII nerve end contacting muscle primordia. **B)** Slit1^−/−^;Slit2^+/−^ with branching errors of nIII and overshooting of muscle primordia. White arrows, axon errors into or overshooting muscles.**C)** Slit1^−/−^;Slit2^+/−^ with similar errors. **D, E)** Slit2^−/−^ and Slit2^ΔMN^ mutants with similar errors. **F)** Quantification of the number of protrusions from the nIII, with loss of Slit2 or Slit2^ΔMN^ causing significant increase in aberrant protrusions (p<0.05, one way ANOVA). Controls (WT and hets), n=5; Slit1^−/−^, n=4; Slit2^−/−^, n=6; Slit1^−/−^2^−/−^, n=4; Slit2^ΔMN^, n=7. **G)** Schematic summary of altered branches and errors. Scale bar. 100μm.

**Figure 8.**
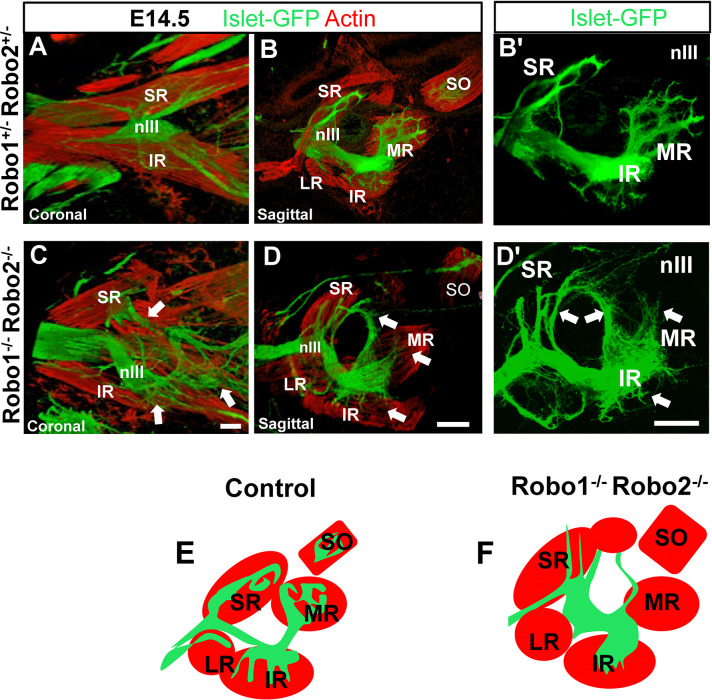
Robo1 and 2 control the innervation of the extraocular muscles. E14.5 embryos (n=4 for each genotype) were labeled for Isl-GFP (green) to visualize nIII axons, and actin (red) for muscles. **A-C)** E14.5, confocal images, maximum projection of nerve and muscle patterns in 400 micron sections. **A)**Coronal view showing the longitudinal axis of the muscles, with compact nerve bundles projecting along the muscle surfaces. **B, B’)** Sagittal sections, showing the penetration and branching of nerves within muscles. No axons were found outside of the muscles. **C, D, D’)** Robo1/2 mutants, with numerous aberrant axon bundles (white arrows) growing in looping, disorganized patterns outside of muscles, including looping around the optic stalk. The inferior rectus (yellow arrow) is contacted by the inferior division but is not penetrated by nIII branches. Similarly, few nerve branches enter the medial rectus. **E, F)** Schematic summary of altered branches and innervation. Scale bar 100μm.

**Figure 9.**
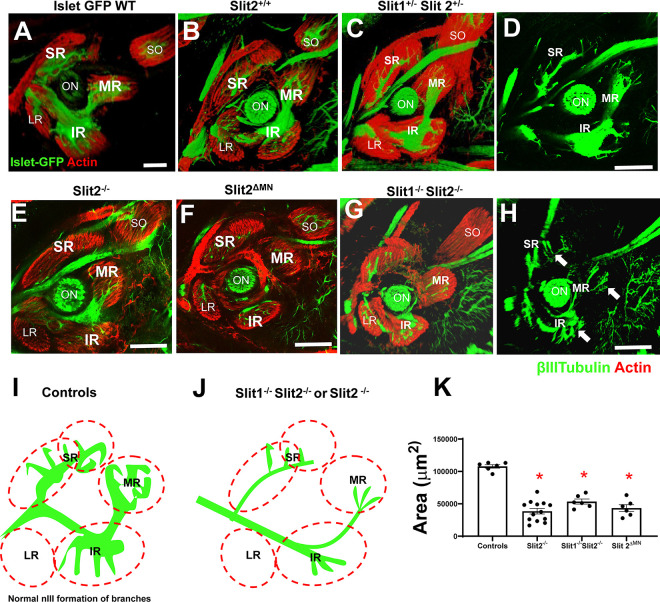
Slit signals required for the innervation of extraocular muscle. E14.5 embryos were labeled for βIII-Tubulin (green) and actin (red). Superior rectus (SR), medial rectus (MR), inferior rectus (IR); Lateral rectus (LR), Optic nerve (ON) and superior oblique (SO) shown as landmarks (see SO and LR analysis in Figures 10 and 11). **A)** Isl-GFP+ control to show nIII projections to muscles. **(A-D)** Several control genotypes were used to visualize the normal pattern of the nIII, with projections into superior rectus, medial rectus, and inferior rectus visible in the confocal planes shown. **E-H)** Slit mutants. Decreased nIII nerve fibers, particularly a smaller inferior division. Branches toward and into muscles are smaller and dispersed (white arrows in H). **I, J)** Schematic summary of altered nerve branches into muscles. (At this stage, the superior rectus had a second division, marked by second dashed oval). **K)** Quantification of area of nIII nerves into extraocular muscles, with loss of Slit2 causing significant decreases. One way ANOVA, * = p<0.05. Controls (WT and hets), n=6; Slit2^−/−^, n=13; Slit1^−/−^2^−/−^, n=6; Slit2^ΔMN^, n=6. Scale bar, 100μm.

**Figure 10.**
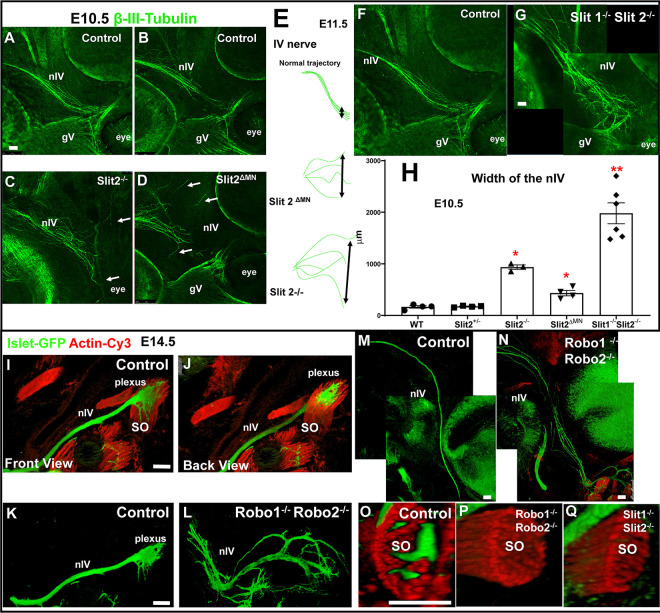
Trochlear nerve (nIV) requires Slit/Robo signals to fasciculate and to innervate superior oblique muscle. Trochlear nerve βIII-tubulin labels on E10.5, 11.5, and 14.5 embryos. These images are different confocal stacks taken from embryos shown in previous figures. **A, B)** E10.5 control nIV, showing axon trajectories toward the dorsal side of the eye. Note that the distal nIV was less fasciculated than nIII. **C, D)** In Slit2^−/−^ and Slit2^ΔMN^ mutants, the axons were defasciculated, with many axons navigating in abnormal directions away from the eye (arrows). **E)** Cartoon of the trochlear nerve phenotypes. The width of the superior and inferior axons of the trochlear was measured (black arrows). **F, G)** E11.5 control and Slit1/2^−/−^ mutant. The altered pattern of the trochlear nerve (nIV) continued on E11.5 with mis-directed trajectories. Similar nIV errors were seen in Robo1/2 mutants (not shown). **H)** Quantification of the increase in nIV width. A statistical analysis was performed comparing several genotypes (n=3–6) (**p<0.01; *p<0.05). **I,J,K,M,O)** E14.5 WT control embryos with Islet-GFP labeling to see the normal pattern of the trochlear. The nerve was cohesive, with a terminus that spread within the superior oblique muscle (SO). Digital rotation was made in 3D images to visualize front (I) and back (J). **L,N,P)** Robo1^−/−^;Robo2^−/−^; Islet/GFP showed defasciculation and mis-directed abnormal branches, with lack of penetration of the superior oblique (SO). **Q)** Slit1^−/−^;Slit2^−/−^ stained for βIII-tubulin showing lack of innervation of SO. Scale bar 100μm.

**Figure 11.**
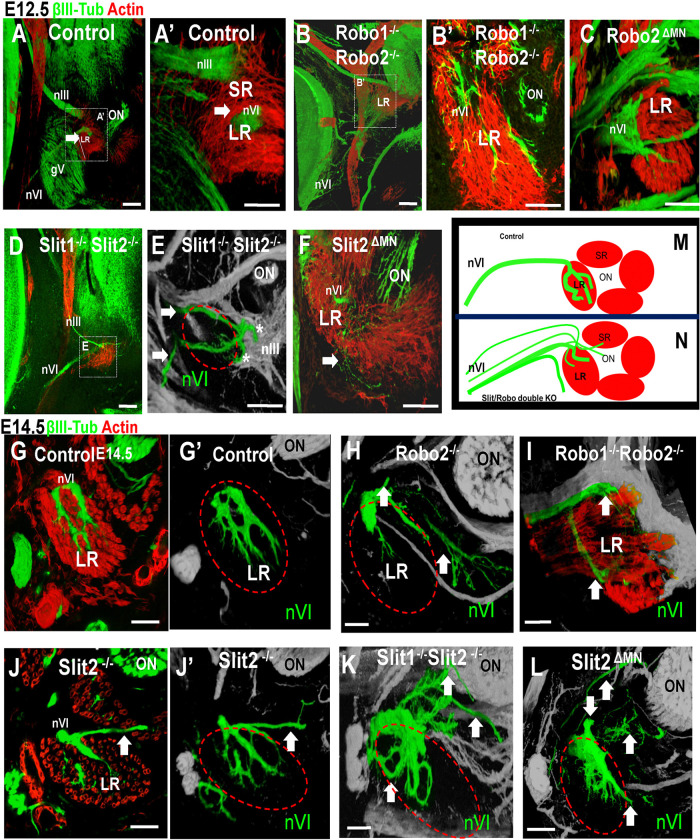
Abducens nerve (nVI) requires Slit/Robo signals to innervate lateral rectus The abducens (nVI) nerve labeled with βIII-tubulin (green) and actin (red). **A)** Control, showing normal projection of nVI at E12.5, terminating at the lateral rectus muscle (white arrow). **A’**) Close-up of A, showing nVI penetration of lateral rectus. **B**,**B’)** Robo1^−/−^; Robo2^−/−^ mutant. nVI is defasciculated, and reaches the primordial extraocular muscles in different position, with only a few axons contacting the lateral rectus (LR). **C**) Robo2^ΔMN^ mutant. nVI axons overshoot the lateral rectus, contacting in an abnormal lateral position. **D)** Slit double knockout, with overshooting and aberrant contact of nVI with nIII. **E)** Closeup of nVI terminus, with color to highlight nVI with abnormal branches that overshoot the muscle target (red outline) and contact (indicated by *) nIII branches (gray, indicated by nIII). **F)** Slit2^ΔMN^ mutant. Few axons penetrate the lateral rectus. **G,G’)** E14.5 control, showing extensive branching within the lateral rectus. **H, I)** E14.5 Robo2^−/−^ and Robo1^−/−^; Robo2^−/−^ mutants similarly show decreased branching within lateral rectus, and abnormal branches overshooting. **J, J”,K,L)** E14.5 Slit2−/−, Slit1^−/−^;Slit2^−/−^, and Slit2^ΔMN^ mutants similarly show ectopic branches, and variable innervation of lateral rectus. **M,N)** Summary cartoon of abducens innervation errors. N= 4–6 of each genotype. Scale bar: 100μm.

**Figure 12.**
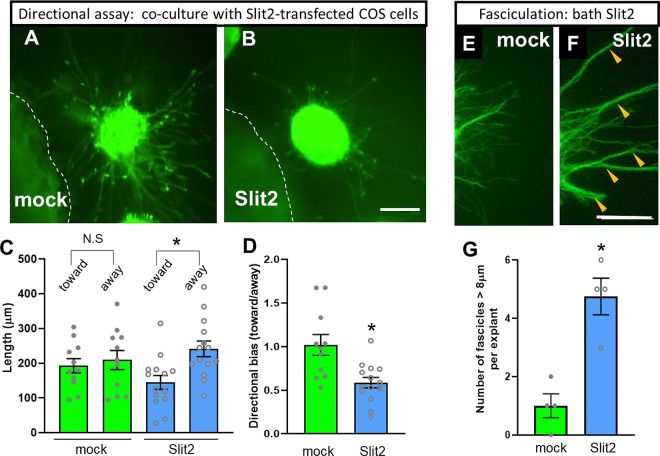
Slit2 both repels and promotes fasciculation of cultured oculomotor axons **A,B)** Explants of Isl1-GFP oculomotor nuclei, co-cultured with aggregates of COS7 cells transfected with no plasmid (A, n=11) or Slit2 expression plasmid (B, n=14) from three independent experiments. Explants were fixed and antibody labelled for GFP to specifically show oculomotor axons. **C)** Quantification of directional axon growth into quadrants toward or away from the COS cells. Average of 10 longest axons. **D)** An outgrowth ratio was calculated by dividing the average axon length in the quadrant toward the cue source, divided by the quadrant away from the cue source. Note the neutral effects of untransfected COS cells, and repulsion by Slit2. **E)** Fasciculation assay, with explants cultured in media conditioned by Slit2-expressing COS cells. Note that in these conditions, Slit2 exposure is non-directional, and increases the number of large fascicles, with longer and thicker bundles (yellow arrowheads). Scale bar: 200 mm. *: p < 0.01 by t-test.

Extraocular muscles are innervated by three nerves: the oculomotor nerve (nIII) which exits the CNS from the ventral midbrain, the trochlear nerve (nIV) that exits at the dorsal midline near the midbrain/hindbrain boundary, and the abducens nerve (nVI) that exits in ventral rhombomere 5. The time course of the oculomotor nerve outgrowth is summarized in Fig. 1A–C, as recently described (Bjorke et al., 2021; Michalak et al., 2017). The main stages of oculomotor nerve development include initial outgrowth to form compact nerves on embryonic day E9.5; a long pause featuring nerve contact with muscle precursor cells from E10.5–12.5, and terminal branching and penetration into differentiated muscles by E14.5. In our study, we first mapped the Slit and Robo expression patterns during these distinct stages (summarized in Fig. 1D,E). We then examined oculomotor nerve development through these stages in Slit and Robo mutants in Figs. 2–9, trochlear nerve in Fig. 10, abducens nerve in Fig. 11, and Slit2 effects on cultured axons in Fig. 12.

### Expression of Slits and Robos during migration of the oculomotor nerves

Previous studies have shown that Slits are expressed in the ventral midline of the mouse neural tube, including the forebrain, midbrain, and hindbrain that flank the oculomotor nerve (Bjorke et al., 2016; Brose et al., 1999; Dugan et al., 2011; Yuan et al., 1999). Furthermore, the oculomotor neurons also express Slit 2 and 3 mRNA, suggesting that the growing axons may also produce Slits as autocrine guidance signals (Bjorke et al., 2016).

We set out to further map Slit and Robo expression along the oculomotor nerve pathway. Slit antibodies that can label Slit proteins in tissue are not currently available. As an indirect marker of Slit expression, we used heterozygous embryos carrying Slit1 and 2 mutant alleles with a tau-GFP reporter insert (Plump et al., 2002), which gave sufficient signal to image cell bodies and tissues that were Slit+, with the advantage over in situ hybridization of also labeling the axons of Slit-expressing motor neurons. Slit1 and 2 GFP labeling overlapped in the ventral midline of the forebrain around through the ventral midbrain and hindbrain, thus flanking the oculomotor and trochlear trajectories as they extended toward the eye during E10.5 (data not shown) and E11.5 (Fig. 1F–G). At the location where the oculomotor nerve approaches the eye to form the plexus, there was not Slit1 or 2 expression in the muscle precursor mass (Fig. 1H). However, there was Slit2 expression within the nearby eye cup. Interestingly, the oculomotor axons were Slit2-GFP+ (Fig. 1H), as were the later trochlear and abducens axons (not shown), consistent with Slit2 mRNA expression in spinal motor neurons (Brose et al., 1999).

We also examined Slit1 and 2 expression around the eye on E14.5 when the motor nerve branches are contacting and innervating the eye muscles. Strong Slit1 and 2 GFP expression was present in a distinct gradient represented in 1E. The gradient seems be from dorsal-high to ventral-low in the mesenchymal tissue around extraocular muscles (green, Fig. 1I’ and J’, two different examples). All the oculomotor nerves were Slit+ (arrows, 1I and 1J). A larger Slit + region filled in the space between the superior rectus, superior oblique and medial rectus, and extended to the Slit + optic nerve (1J and J’). Interestingly, the extraocular muscles themselves were negative for Slits as shown by unlabeled spaces in 1I’ and J’.

We also examined Robo expression by oculomotor axons. In previous studies, we showed that Robo 1 and 2 are expressed in the nIII nucleus, by both in situ hybridization and Robo antibody labeling (Bjorke et al., 2016). We extended this by examining Robo expression in extending oculomotor axons using Robo1 and 2 antibody labeling on E11.5 and E14.5. On E11.5 when beta-tubulin positive axons are navigating to the eye, we found Robo1 and 2 in the oculomotor nerve extending to the plexus (Fig. 1K, L), and similar labeling in the trochlear and abducens (not shown). At E14.5, the oculomotor nerve was labeled for both Robo1 and 2, but appeared only in the inferior branch (white arrows, Fig. 1M, O), extending to both IR and MR muscles. The superior branch axons did not appear to express Robo1 (yellow arrows, 1M and M’). Overall, we find Robo expression in the oculomotor nerve navigating to the eye, as well as Robo expression in the developing extraocular muscles. Robo expression in motor nerves, particularly in early extending axons, suggested a function in Slit-mediated guidance.

### Slit/Robo control fasciculation of the oculomotor nerves.

To test the function of Slit/Robo signals in guiding the oculomotor nerves, we started by examining oculomotor nerve development on E10.5 in Robo1/2 double mutants. By E10.5, a tightly fasciculated oculomotor nerve has grown out to the eye (Fig. 2A), with more axons added by E11.5 (Fig. 2H). A view of the oculomotor nerve at a higher magnification on E10.5 in wild type as well as in Robo1^+/−^;2^+/−^ heterozygotes reveals that a few axons are not completely cohesive with the primary nerve (yellow arrow, 2A’, 2B’), but no axon tips can be seen outside of the nerve. In contrast, Robo1^−/−^; Robo 2^−/−^ double mutants had many defasciculated axons, with smaller fascicles as well as axons navigating away from the main nerve (Figure 2C, D).

In Robo double mutants, the nerve formed several fascicles independent from the leading fascicle (Figure. 2C, 2C’, blue arrows), as well as multiple single axons (Figure. 2C’, 2D’, yellow arrows). The defasciculated axons diverged from the normal trajectory, with some diverging sharply off track, while others grew roughly in the correct direction toward the eye. Misguided axons or fascicles were shorter than the longest axons that stayed on course to reach the plexus. The nerve bundle was smaller distally, likely due to the lost axons. The axon terminations in the plexus were also disorganized (Fig. 2C”). We found a significant difference between heterozygous controls and mutants in the formation of fascicles and axons navigating alone (Figure. 2F and 2G).

At E11.5, the Robo1/2 double mutants had increasingly severe defasciculation errors, failing to recover the disruptions (Fig. 2I and I’), compared to the single cohesive nerve in wild type (Fig. 2H–H’). Many smaller fascicles formed, and these tended to project out toward the eye, suggesting that navigational cues were sufficient, even while fasciculation was disrupted.

### Lack of Slit1 and 2 alters fasciculation of nIII

To test the requirement for Slits in the initial nerve outgrowth, we compared control littermate embryos, wild type or Slit1^+/−^; Slit2^+/−^, with Slit1^−/−^; Slit2^−/−^ double mutants Considerable numbers of axons defasciculated from nIII in Slit double mutants, similar to Robo1/2 double mutants (Fig. 3B, F). In E10.5 Slit1^−/−^; Slit2^−/−^ double mutants, we observed significant numbers of axons forming aberrant fascicles and axons navigating independently (Figure. 3B, C, D). On E11.5 in wild type, the nIII bundle stops near the eye to form a compact plexus (Fig. 3E). In Slit double mutants, the nerve end remained defasciculated (Fig. 3F, yellow arrows), with significant errors compared to controls (Fig. 3G).

The similarity of the nIII fasciculation errors between the Robo and Slit mutants is consistent with Slit 1 and 2 acting through Robo receptors to promote a cohesive oculomotor nerve.

### Lack of Robo 2 in motor neurons disrupts the nIII plexus

The prominent expression of Robo1 and 2 in the oculomotor nerve is consistent with direct function within this axon population. To begin to determine the function of Robo in nIII, and to distinguish between Robo1 and 2 functions, we deleted Robo2 in motor neurons using a Robo2 floxed allele, crossed with a motor neuron-specific Islet-Cre line (Lewcock et al., 2007). This allowed us to remove Robo2 specifically in motor neurons.

We generated several genotypes from these crosses, which included controls, Robo2^ΔMN^ deletions, and Robo2^ΔMN^ deletion combined with Robo1 constitutive knockout. Controls at E10.5 had a single compact nerve ending in a compact plexus near to the eye (Figure. 4A). In contrast, Robo1^−/−^; Robo2^ΔMN^ showed strong defasciculation (Fig. 4B), similar to the Robo double mutants (Figure. 2). The oculomotor nerves in Robo1^−/−^;Robo2 ^ΔMN^ mutants dispersed the plexus to distribute widely away from the eye and toward the hindbrain. This shows that, in a Robo1 mutant background, Robo2 has an autonomous function in motor axons. Interestingly, when we depleted Robo2 function in motor neurons while retaining wild type Robo1 function (Robo1^+/+^;Robo2 ^ΔMN^), the nerve was fasciculated but the plexus spread abnormally (Fig. 4C). Because the plexus normally forms by the axons spreading out in contact with a restricted sub-region of a Pitx2-expressing precursor mass, including muscle precursors, we cut thin sections to compare the plexus fibers with the precursors marked by Pitx2 antibody, and Isl-GFP motor neuron marker to verify specific nIII plexus fibers. In Robo2^−/−^ mutants, the Isl-GFP + nIII fibers were more widely spread throughout the Pitx2 + precursor mass, and therefore contact precursors outside of the normal sub-region (Fig. 4D–E). At high magnification (4Dꞌ), the plexus was larger in Robo2^−/−^ or Robo2 ^ΔMN^ mutants (Fig. 4G–H), with significantly larger area (Fig. 4J). Thus, Robo1 and 2 cooperate with redundant functions to promote oculomotor nerve fasciculation, while Robo2 has an independent and motor neuron-autonomous function in the nerve to stop growth to make a restricted plexus.

### Slit 2 expression by oculomotor axons is required for correct navigation of nIII

With the aim to test the function of Slit2 expressed by the oculomotor neurons, we crossed Slit2^flox^ with Islet1-CRE. This cross produced Slit2 ^ΔMN^ mutants lacking Slit 2 in motor neurons, as previously characterized (Kim et al., 2019). On E10.5, the nerve remained fasciculated, but the position of the plexus shifted away from the eye, similar to Slit2 global homozygous mutants (Fig. 5A–C). To quantify the distance between the plexus and the eye, we measured the distance between the tip of the longest axons and the optic fissure. The distance of the axons to the optic fissure was significant farther in both Slit2 mutants and Slit2 ^ΔMN^ (Fig. 5D–F). At E11.5, the plexus of the nIII was also strongly altered in Slit2 ^ΔMN^, with axons overshooting the plexus to project around the eye (Figure. 5H–L), prematurely by about a day, again similar to Slit2 global homozygous mutants (Fig. 5J). Overall, the function of autocrine Slit2 produced by nIII axons may modify how motor axons interact with their intermediate target tissue, with autocrine Slit2 required for a stop signal to prevent the premature growth of axons around the eye.

### Contact of nIII with the extraocular muscles depends on Robo and Slit

On E12.5, the extraocular muscles begin to differentiate to form organized actin-positive fiber bundles around the eye. Following muscle organization, axons from nIII grow away from the plexus to initiate the nerve branches, and along with nIV and nIV, begin to contact their muscle targets as shown in Fig. 1. To test the functions of Slit and Robo in the formation of the major branches and initial muscle contact of the oculomotor nerves on E12.5, we analyzed several combinations of Robo1/2 and Slit1/2 mutations (see Figs. 6 and 7).

On E12.5, Robo1/2 double mutants showed considerable defasciculation of nIII, similar to earlier stages. Many of the axons reached the area between the extraocular muscles and the optic stalk; nevertheless, axons shot outside the muscles and generated aberrant protrusions, including spreading around the optic stalk (Fig. 6D–I, arrows in Figure. 6E, H, F). Some axon bundles traversed across the muscles out into adjoining tissues, failing to stop and penetrate the muscles. A similar pattern happened in Robo1−/− Robo2 ^ΔMN^, indicating that the Robo requirement was autonomous to the motor axons (Fig. 6G–I). Robo double mutant protrusions had twice as many bundles, but interestingly, in Robo1^−/−^Robo2 ^ΔMN^ the defasciculated axons formed four times more bundles than controls (Fig. 6J). Overall, the mutant axon patterns included at least three types of errors, as summarized in Fig. 6K: defasciculation into numerous small ectopic branches, aberrant projections of many ectopic branches with overshooting into areas consisting of Slit + mesenchyme, and failure of some axon bundles to stop in contact with the primordial muscles.

To examine the function of Slit proteins at E12.5, we analyzed several Slit mutant combinations (Fig. 7). We found that Slit1^+/−^Slit2^+/−^ and Slit2^+/−^ heterozygous embryos showed normal patterns of nIII axons (Fig. 7A). Slit1^−/−^ Slit 2^+/−^ mutants showed a few axons projecting outside of the extraocular muscles (7B, white arrows). Slit2^−/−^, Slit1^−/−^Slit2^−/−^, and Slit2 ^ΔMN^ all had significant errors. The axon errors were similar to Robo1/2 mutants, and included defasciculated bundles that spread out from the main inferior branch, and aberrant projections around the optic stalk across muscle primordia. The axon protrusions overshooting were significantly increased in Slit2^−/−^ or Slit2 ^ΔMN^. The number of nIII bundles was also significantly increased in Slit 2^−/−^, Slit1^−/−^Slit2^+/−^, Slit1^−/−^Slit2^−/−^, and Slit2 ^ΔMN^ mutants (Fig. 7G). The spreading and aberrant axon bundles in Slit2 mutants suggest an important function for Slit2 in restricting axon projections to large bundles, possibly by repulsion from Slit2-expressing mesenchyme around the muscles. However, motor axons also require Slit2 expression as an axon-derived autocrine signal.

### Penetration of the extraocular muscles depends on Slit/Robo signaling.

The pattern of innervation of the eye muscles is essentially complete by E14.5, so we examined this stage to see the effects of Robo or Slit loss. In control embryos, the oculomotor nerves had clear branches that projected to and penetrated each muscle (Fig. 8A–C). The spreading of axons into the muscles was similar for each muscle, consisting of a few thick sub-branches that spread within the muscle midpoints. Robo1/2 double mutants had abnormal branches around the muscles (Fig. 8C–D). Compared to the overshooting axons on E12.5, most were apparently retracted. The inferior rectus and medial rectus consistently lacked innervation (Fig. 8D). Thick bundles of nIII axons looped up into the superior orbit in double mutants (Fig. 8D and D’, red arrow), retaining these errors from E12.5. The normal innervation of the medial rectus was absent (Fig. 8D’, white arrow), with just a few axons reaching the muscle. Axon branches did approach the superior rectus, but most of them remained outside of the muscle (Fig. 8D’, blue arrow). Particularly, the inferior branch of the nIII Robo1/2 double mutants (Fig. 8, yellow arrow) showed a lack of deep penetration of the inferior rectus, with just a few protrusions coming from the primary branch (8D and D’). Overall, although many oculomotor axons approached the eye muscles, the axons generally failed to penetrate the muscles, with only a few abnormally organized bundles in the superior and inferior rectus muscles.

We also noted that Robo1/2 mutant embryos had altered muscles on E14.5. The muscle fibers appeared to be less compact, with small gaps between fibers. We also noticed that blood vessels around the muscles were disrupted or not present in Robo double mutants, although we did not further analyze. Therefore, Robo1/2 may have additional functions in the muscle fibers and blood vessels.

Slit mutant embryos also had strong errors in oculomotor nerve branches and innervation on E14.5, using the Isl-GFP motor axon marker (Fig. 9). Similar to Robo1/2 mutants, Slit mutant nerve branches generally were defasciculated and disorganized, but with less misdirection and overshooting. We found that in the absence of Slit2, the pattern of innervation was similar to the controls, but the thickness of the nerve branches was diminished. The area covered by axons within the muscles was significantly reduced in the various Slit mutants (Fig. 9K), consistent with axons failing to penetrate the muscles (Fig. 9F–K).

### Slit/Robo controls the fasciculation and navigation of the nIV toward the superior oblique muscle.

We also tested whether other motor nerves required similar Slit/Robo guidance functions. The trochlear nIV nerve projects first within the dorsal part of the hindbrain to exit the CNS at the midbrain/hindbrain boundary, then projects laterally toward the eye. On E10.5, trochlear navigation in controls is fairly defasciculated, but tends toward the dorsal eye (Figure. 10A, B). We analyzed Slit2 mutants, including Slit2^−/−^ and Slit2 ^ΔMN^ (Figure. 10C, D), as well as Slit1^−/−^Slit2^−/−^ (Figure. 10G). In all of the Slit mutants, the trochlear axons were more spread out than in controls, with defasciculation and diverging trajectories seen with Slit2 loss either globally or in motor axons, with Slit1^−/−^Slit2^−/−^ mutants most severe (Fig. 10H). On E11.5, this defasciculation was maintained in Slit2^−/−^ embryos, and Robo1/2 mutants showed similar defasciculation at the early stages of E10.5 and 11.5 (data not shown).

To examine the later stage of innervation of the superior oblique muscle, we analyzed Robo1^−/−^2^−/−^ embryos on E14.5. Normally, in wild type Islet-GFP embryos, the trochlear nerve was condensed (Figure. 10I–K and M), until the axons spread out on contact to penetrate the superior oblique (SO) muscle (Fig. 10J and O). In Robo1/2 mutants, the trochlear nerve formed ectopic branches which were numerous and misguided, covering a wide area around the dorsal eye (Fig. 10L, N). Importantly, 3D reconstruction analysis of confocal sections along the length of the muscle in Robo1/2 or Slit1/2 double mutants showed that no axons contacted or penetrated the superior oblique muscle (Fig. 10O, P, Q), suggesting a specific Slit/Robo requirement for muscle innervation. We noted that some trochlear axons formed branches near the superior rectus, possibly initiating abnormal innervation. Therefore, similar to the oculomotor, the trochlear nerve requires Slit/Robo signals at several steps during its trajectory toward the eye to organize fasciculation, and final approach and contact with its muscle target.

### Severe alterations of the abducens nerve in Slit/Robo double mutants

The abducens nerve (nVI) emerges from rhombomere 4 in the hindbrain and navigates in an ascending trajectory to the lateral rectus muscle (Fig. 11A and A’). We analyzed the abducens in several mutant combinations for Slit1 and 2, and Robo1 and 2. We found that in the absence of Robo1 and 2, the abducens was defasciculated (Fig. 11B and B’), and similarly in Robo2 ^ΔMN^ (Fig. 11C). In Slit1/2 and Slit2 ^ΔMN^ mutants, we observed defasciculation and an altered pattern of navigation (Fig. 11D–F). The main abducens fascicle normally avoided contact with the oculomotor nerve, but the abducens nerve in mutants came into close contact with the oculomotor nerve. On E14.5, the wild type abducens penetrated the lateral rectus, forming two principal bundles, which further split into smaller branches (Fig. 11G and G’). In Robo1/2 mutants, the abducens overshot outside the lateral rectus (arrows, 11H) and just a few axons penetrated the correct target (Figure. 11H, I). Likewise, in Slit2 mutants, several fascicles projected outside of the lateral rectus (Fig. 11J, K), with similar errors in Slit2 ^ΔMN^. In Slit1/2 and Robo1/2 double mutants, abducens made stronger errors, splitting into more branches, with branches outside of the target muscles forming loops, and projecting near the optic nerve and other muscles (Fig. 11H and L). Overall, the abducens axons made navigational errors in Robo and Slit double mutants, with only a subset of the axons reaching and penetrating their target muscle (summarized in Fig. 11M,N). These errors were similar to oculomotor and trochlear nerves, suggesting parallel Slit/Robo functions in promoting nerve fasciculation, guidance of nerves to muscles, and targeting nerve growth into muscles.

### Slit2 repels and stimulates fasciculation of oculomotor axons.

A shared disruption in the oculomotor nerves in Slit and Robo mutants, in both early (E10.5-E11.5) and late (E14.5) stages, was defasciculation of the nerves. Defasciculation could be seen early, as the main bundle split into smaller bundles and single axons, and later, as nerve branches around the eye formed smaller and mis-directed bundles. While the Slits have the canonical function as chemorepellents, Slit2 can also increase the fasciculation of cultured phrenic motor axons (Jaworski and Tessier-Lavigne, 2012). To test for direct effects of Slit2 on oculomotor axons, and to test if Slit2 acts to repel or promote fasciculation, or both, we developed culture assays for oculomotor explants in collagen gels. Ventral midbrain tissue containing Isl1-GFP oculomotor axons was dissected on E11.5 when the motor nerves are actively growing and condensing into nerves.

To test Slit2 effects on oculomotor axon directional growth, we co-cultured oculomotor explants with COS cell aggregates that were transfected with full length Slit2 (Fig. 12A–D). In control explants exposed to an aggregate of mock-transfected COS cells, the oculomotor axons grew out in a symmetric radial pattern. The axon outgrowth in the quadrants toward or away from the COS cells was not significantly different (Fig. 12A, C, D). However, the axon outgrowth was biased significantly away from Slit2-transfected COS aggregates (Fig. 12B, C, D), demonstrating that oculomotor axons have a chemorepulsive response to Slit2 in culture, consistent with prior chemorepulsion assays (Hammond et al., 2005).

To test Slit2 effects on oculomotor axon fasciculation, we cultured oculomotor explants with non-directional Slit signals. Oculomotor axons growing out from explants with bath application of conditioned media from Slit2-transfected COS cells formed more large axon bundles (> 8 microns) than media from mock-transfected COS cells (Fig. 11E–G). Unexpectedly, we also noted that the oculomotor axon outgrowth appeared to be stimulated in these culture conditions. The increased fasciculation is a positive response to Slit2 that is consistent with the decreased fasciculation and associated axon wandering errors in Slit and Robo mutant oculomotor nerves.

Together, the results from cultured oculomotor axons show both chemorepulsive and fasciculation responses to Slit2 signals, consistent with the in vivo guidance roles suggested by the analysis of Slit and Robo mutants.

## Discussion

**Figure 13.**
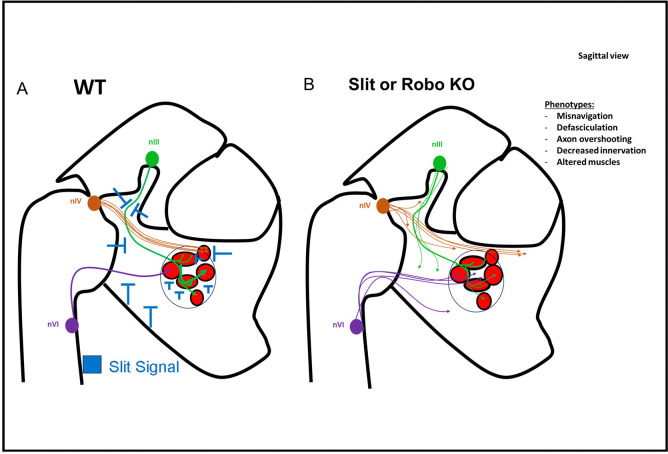
Slit controlling navigation, fasciculation, branching and connectivity of the oculomotor nerves Summary of oculomotor nerve projection patterns in control (A) and in Robo and Slit mutants (B). A) In Slit+ control embryos, Slit signals (blue repulsion symbols) come from the flanking CNS ventral midline tissues, from mesenchymal tissues surrounding the eye muscles, and from the motor axons. In Robo and Slit mutants, the initial motor nerves were defasciculated. The nerves navigated along abnormal trajectories as they approached the eye. Later, near the eye muscles, the nerves overshot the muscles, with decreased contact with muscles and decreased or absent penetration of muscles.

The main findings are that Slit/Robo signals control the navigation and connectivity of the oculomotor nerves during mouse embryo development. Overall, our study finds that Slit/Robo signals are re-used in distinct ways at several decision points in oculomotor development (Fig. 13). These steps include initial outgrowth of cohesive nerves, stopping to form a plexus in contact with Pitx2 + precursors, precise formation of branches, and finally contact and penetration to innervate eye muscles. Significantly, a subset of these navigation decisions depends on cell autonomous production of Slit2 by oculomotor axons. Some of these Slit/Robo guidance functions are consistent with conventional Slit chemorepulsion, while other phenotypes implicate Slit/Robo dependent axon fasciculation, which we find can act directly on cultured oculomotor axons. We discuss these diverse Slit/Robo functions in turn, considered in the context of what else is known about the guidance of motor nerves, with particular implications for oculomotor system development.

### Slit/Robo signals organize initial motor nerves by promoting fasciculation.

The bundling of motor nerves enables a cohesive cohort of motor axons to efficiently reach major decision points. A major finding of our study was that Slit/Robo signaling is critically required to organize the pioneer oculomotor axons into a single large bundle. In the absence of Robo1/2 receptors (Fig. 2), the axons failed to fasciculate, and instead diverged to navigate independently or to generate smaller aberrant bundles. We found similar defects in Slit1/2 mutants (Figure. 3), where axons defasciculated and were detached from the leading axons. We observe defasciculation early, from the midbrain exits, as well in the last steps of nerve branches approaching the muscles, suggesting that this Slit/Robo requirement is continuous through motor nerve pathway formation. Similar motor nerve defasciculation in Slit and Robo mutant embryos was previously seen for the phrenic motor nerve, with premature splitting as the nerve fibers approach their diaphragm muscle target (Jaworski and Tessier-Lavigne, 2012).

Slit signals could increase fasciculation using multiple mechanisms. Considering classical Slit chemorepulsion for axons, the oculomotor nerve projections are flanked by the Slit + ventral midbrain/hindbrain and ventral forebrain, providing surround repulsion to condense the nerve. The splitting of the oculomotor nerve fascicle in Robo1/2 mutants is consistent with reduced surround repulsion. Similarly, our previous studies of the initial segments of spinal motor nerves also reveal wider exit points, suggesting a general role for Slits in promoting motor nerve fasciculation (Kim et al., 2015; Kim et al., 2019).

Slit signals may also act positively to promote motor axon fasciculation. Bath application of Slit2 increases the bundling of phrenic motor axons (Jaworski and Tessier-Lavigne, 2012). We find a similar effect of Slit2 increasing large fascicles of oculomotor axons in culture (Fig. 12). This Slit function to promote axon fasciculation may occur through an autocrine mechanism, previously implicated by Slit expression in motor neurons, Slit effects on cultured axons, and global Slit knockouts. To test for an in vivo autocrine Slit mechanism, we used conditional genetic deletion of Slit2 specifically in motor neurons, and found later stages of nerve bundles were disrupted. This positive pro-fasciculation of Slit2 likely acts cooperatively with other guidance factors, because mutations in other genes also cause defasciculated motor nerves. For example, Ephrin A and Ephrin B guide motor innervation in the lateral and medial motor columns in chick limb (Dudanova et al., 2012). The Sema receptors Npn1/2/PlexinA, and knockdown of Plexin A1 or the cytoplasmic Sema mediator α-Chimaerin causes nIII and nIV defasciculation near the eye muscles (Ferrario et al., 2012). Robo function may be directly involved in axon fasciculation through its capacity as a cell adhesion molecule, or could interact with other CAMs such as Tag-1, DCC, NCAM and L1 because their similarities with the Ig-super family (Liu et al., 2004). An alternative direct mechanism is Slit/Robo signals acting via the Down Syndrome cell adhesion molecule DSCAM. Drosophila DSCAM binds to Slit, and DSCAM can form a complex with Robo to increase the fasciculation and growth of longitudinal axons in the fly nerve cord (Alavi et al., 2016). Resolving in future experiments which molecular mechanisms promote motor axon fasciculation will be important to understand how populations of motor axons interact with each other to efficiently project to target tissues.

### Slit controls navigation of oculomotor nerves to contact an intermediate target next to the eye by motor axon-derived Slit signals.

The trajectory of the oculomotor nerve is normally straight to the ventrolateral side of the eye, where the axons spread out slightly in a plexus. Slit1/2 and Robo1/2 mutants make nIII errors of diverting away from the eye, and overshooting the plexus, revealing two potential functions of Slit/Robo signals. Significantly, both of these guidance steps were also strongly disrupted in Slit2^ΔMN^ embryos, implicating motor neuron-derived autocrine Slit2 signals in this guidance. For setting the trajectory to the eye, nearby external Slit sources are the flanking hindbrain ventral midline, and the optic vesicle. The Slit2^ΔMN^ errors implicate autocrine Slit2 functions in modulating axon responses to cues in this area. The later overshooting nerve errors implies either a reduced sensitivity to Slit2 repulsive signals that prevent premature axon projections around the eye, or a loss of a Slit2-dependent adhesive affinity for the Pitx2 + precursors causing a failure of axons to pause and spread to form the plexus. The mechanisms for Slit2 autocrine production, and their effects on growing axons, are unknown, but trans-axonal signaling is recognized in diverse axon guidance behaviors (Spead and Poulain, 2020), including organization and patterning of target tissues (Caipo et al., 2020). This could involve either increasing or decreasing the effects of externally-derived Slit2, or modulating axon responses to other guidance cues (Dupin et al., 2015).

### Disruption of oculomotor nerve branching to eye muscles

The terminal “branches” split off from the main nerve bundle by selective defasciculation of axon subpopulations to grow to their target muscles. Terminal branch formation was highly disrupted in Slit and Robo mutants, with numerous ectopic branches navigating abnormally within the eye region, in between and past muscles, and around the optic stalk. Slit + mesenchymal tissues positioned around the eye muscles may act as repulsive boundaries channeling nerve branches to the muscles. The earlier plexus errors also likely account for some of the terminal branch errors. On the other hand, the similar branch errors in Slit2^ΔMN^ mutants indicate that motor axon Slit2 production is also required for terminal branch navigation. The trochlear and abducens nerves make similar errors, consistent with parallel Slit/Robo functions. Together, the branch errors show that Slit/Robo function is required to guide selective defasciculation from the main bundle, then later influence the targeting to muscles based on muscle-specific attractive or repulsive cues.

### Decreased eye muscle innervation in Slit and Robo mutants

As terminal branches extend to muscles, the axon bundles normally contact their target muscle at specific regions. The axon bundle then penetrates between muscle fibers, with axons dispersing for eventual monosynaptic innervation of each muscle fiber. In Slit and Robo mutants, motor axon entry into muscles on E14.5 was reduced. The earlier errors likely reduced the number of axons that approached muscles. Other axons grew near but did not penetrate muscles. Those axons that entered muscles had abnormal branching patterns, and did not extend as far within the muscles. The trochlear and abducens nerves provide clear evidence for reduced innervation and mis-targeting that was not possible to distinguish for the several branches of the oculomotor nerve. We also noted that Robo mutants had abnormal muscle fibers, with disrupted or delayed adherence of muscle fibers. In developing muscles in Drosophila, Slit and Robo mutations disrupt myotube guidance and muscle fiber interactions (Chanana et al., 2009). The early abnormalities in Robo mutant muscle fiber organization in eye muscles may represent similar functions in muscle cell differentiation or adhesion.

Currently, Slit/Robo signals have not been directly implicated in congenital cranial dysinnervation disorders in humans, aside from the divergent Robo3 receptor which does not bind Slits (Zelina et al., 2014). However, the results from the Slit and Robo mouse mutants described here provide models for a wide range of developmental defects in the oculomotor system. The range of nerve errors, reduced and mis-innervation, and abnormal muscle formation suggests that partial or localized perturbation of Slit/Robo signaling has the potential for functional disruption. However, identification of altered eye movements in mice would require conditional knockout strategies due to the lethality of Slit and Robo mutations. The requirement for Slit/Robo guidance at multiple steps also suggests that Slit/Robo signals cooperate with other guidance molecules to ensure effective innervation of the extraocular muscles.

## Conclusions

This study has identified multiple functions for Slit/Robo signaling in guiding several steps of oculomotor nerve development and innervation of extraocular muscles.

## Data Availability

The datasets used and/or analyzed during the current study, and the materials generated in this study are available from the corresponding author on reasonable request.
